# On Some Manifestations of Scorbutic Disease in Children

**Published:** 1894-06-30

**Authors:** G. A. Sutherland


### Medical Progress and Hospital Clinics.

[*The Editor will be glad to receive offers of co-operation and contributions from members of the profession. All letters should be addressed to* The Editor, The Lodge, Porchester Square, London, W.]

### ON SOME MANIFESTATIONS OF SCORBUTIC DISEASE IN CHILDREN.

By G. A. Sutherland, M.D.Edin., M.R.C.P.Lond.

In the case of every disease, as our knowledge of it gets defined, there comes to be recognised a series of symptoms which outline the course of a typical case. Whilst an acquaintance with these cardinal symptoms and the order of their appearance is necessary for the practitioner, it is also misleading unless the aberrant or irregular forms of the disease are also understood and recognised. In this paper I shall first of all relate a typical case of infantile scurvy, and then describe some of the more unusual forms and complications met with in children.

Case I.—A female child, aged sixteen months, was admitted to hospital on May 15th, 1892. Her food, since she was three weeks old, had been condensed milk with occasionally a piece of bread. Two months previously her gums had become swollen, and had been lanced by a doctor. The child gradually became restless and fretful, then tender to touch, and for a fortnight before admission she had screamed violently whenever she was moved, and her sole desire seemed to be to lie perfectly still. She would remain for hours quite apathetic, lying on her back with the thighs flexed on the abdomen. On admission her condition was as above described. The thighs and legs were tense and shiny in appearance, and both ankles were swollen and œdematous. Numerous hæmorrhages were scattered over the trunk and extremities. The gums were swollen, and spongy, and bled easily. No teeth were visible, and the mother stated that none had appeared. Rachitic changes were well marked. A diet of cow's milk, milk puddings, and oranges was ordered. Four days later distinct improvement was noted. The gums were less swollen, and the tenderness in the limbs had diminished. On the sixth day the child could move her limbs without pain, and there was very little œdema of the extremities left. She was discharged on the tenth day, the only trace of the disease left being a certain amount of anæmia. The signs here—spongy gums, sub-periosteal effusion in the thighs and legs, with œdema round the ankles, and skin hæmorrhages—taken along with the history of a diet from which all fresh food was absent, and the rapid recovery on the administration of fresh milk and oranges, clearly prove the case to have been one of scurvy.

One of the symptoms of scurvy is aching in the limbs, and if this is unaccompanied by any other marked signs of the affection, the disease is apt to be looked on as rheumatic.

Case II.—A female child, aged five years, was seen in October, 1889. She had been ill for one month, and had lost the power of standing or walking. She complained of pain in the limbs and was extremely tender to touch. The tonsils were inflamed and enlarged, and a systolic murmur was present at the apex of the heart. I diagnosed the case as one of rheumatism, and ordered a mixture containing salicylate of sodium. She was treated at home for a month for acute rheumatism, and then, as she seemed to be getting worse, the doctor in attendance sent her again to the hospital, and she was admitted. The following history as to her diet was then obtained. During the previous six months she had lived entirely on bread and butter, condensed milk, tea, and occasionally a little meat. All her life she had had a positive aversion to fruit or vegetables of any kind. The child was found to be suffering from pneumonia, affecting the upper lobe of the right lung. She had a marked disinclination to move or even to speak, and cried when her limbs were touched. She lay in bed with the knees and elbows flexed. The gums were healthy, there were no skin hæmorrhages, and no œdema, but a distinct thickening could be made out along the course of the left tibia. Some ulceration was present on the labia majora. The urine contained a small amount of albumen. She died ten days later, the inflammation of the right lung having spread through its whole extent. At the necropsy, in addition to the evidence of pulmonary disease, the periosteum of the left tibia was found to be detached over the whole diaphysis, and the bone was white and necrosed in appearance. A layer of semi-solid blood clot lay between the bone and the periosteum. The lower epiphysis was separated from the shaft.

This case illustrates one of the commonest and one of the most fatal complications of scurvy—namely, pneumonia. The onset of scorbutic symptoms was misleading, although, if the history as to diet had been inquired into when the patient was first seen, it is probable that the mistaken diagnosis of rheumatism would not have been made. The complete absence of any signs of gingival affection was certainly unusual in a child of five years, but serves to emphasise the fact that although hæmorrhages or fibrinous effusions are always present in scurvy, no rules can be laid down as to the exact localities in which they will first appear. The form above described, in which the patient complains of pain in the limbs, tenderness on pressure over the muscles, and possibly loss of the power of standing or walking, is not uncommon. Attacks of this nature, due to subperiosteal effusion, may pass off, especially during the summer months when fruit and vegetables are plentiful, and then recur again in the winter. These repeated attacks lead to the formation of nodes on the long bones due to localised periosteal hæmorrhages, and in other cases to wasting of the shaft of the bone, its blood supply having been cut off by the detachment of the periosteum. The effusion sometimes manifests itself around the muscles or their tendinous attachments, and rigidity of the knee or ankle joint may supervene, the knee becoming flexed and the foot pointed as organisation and contraction of the effused material proceed.

A tendency to cardiac syncope is in some cases of scurvy a prominent symptom, and one of grave importance. Minor attacks of this nature are not uncommon, but are usually recovered from under rest and stimulation. The occurrence of sudden cardiac failure in the case of adults suffering from scurvy is referred to by the chief authorities on the subject (Lind, Budd, Buzzard, and Hilton Fagge). As regards children, the danger has not yet been recognised, and I have not met with any record of a fatal result, as in the following case.

Case III.—A boy, aged four years, was brought to hospital for the treatment of sores on his fingers. His mother stated that he had been perfectly well until six days previously, when she noticed a blister forming on his right forefinger and another on his left middle finger. These blisters had broken down and ulceration had followed. He had also suffered from epistaxis for a few days. The child was very anæmic and showed signs of advanced cachexia. As the fingers were being dressed he moaned a good deal in a subdued manner. Further examination showed that the nostrils were filled with dried blood, and that numerous hæmorrhages were present over the trunk and extremities. Some of these were small, and others were as large as a two-shilling piece. The ulcers were situated at the roots of the fingers, had an unhealthy sloughing appearance, and the skin for some distance around them was dark in colour and looked gangrenous. The patient's diet had been chiefly bread and butter and tea. Milk he would not take, but he had occasionally meat, potatoes, fish, and eggs. As the child was manifestly suffering from some constitutional affection, his mother was advised to leave him in the hospital. She retired to the waiting-room to consult her friends on this subject, and a few minutes later came running back saying the child had a fit. He was breathing stertorously and very slowly, the pulse was not perceptible, nor could the heart sounds be heard. After a few more breaths the patient expired. The necropsy did not throw much further light on the case. There was some extravasation of blood beneath the pericranium without any external signs of bruising. The brain presented no evidence of disease. The left ventricle of the heart was in a condition of almost complete diastole, and both ventricles were empty. In connection with this case I attach great importance to the profound cachexia from which the child was evidently suffering. This is usually well marked in advanced cases of scorbutic disease, and yet is so gradual in its development that, as in the case recorded, it may not attract the notice of the patient's relatives. Fainting has been described by Dr. Barlow, and others, as not uncommon in scorbutic disease in children, and it is but a step further to fatal cardiac syncope, such as was the cause of death in this case.

While certain cases present symptoms simulating those of rheumatism, there are others in which a diagnosis of syphilitic epiphysitis is frequently made. Subperiosteal hæmorrhage in scurvy may commence at one of the epiphyses of a long bone,—one side being usually affected for some time before the other—and an extremely tender swelling is then found around the end of the femur or the tibia, and the limb is apparently paralysed. The condition is very similar to that described by Parrot as "syphilitic pseudo-paralysis." As other important symptoms, such as anæmia, cachexia, separation of the epiphyses, and hæmorrhages are also common to both syphilis and scurvy, the diagnosis may be extremely difficult. The syphilitic affection of bone occurs usually in infants under six months of age, while scurvy is rarely found until after that age. In doubtful cases an exploratory puncture may be made, when the presence of pus in the former, and of blood in the latter disease will clear up the diagnosis.

The most important diagnostic method in all cases is the administration of fresh fruits and vegetables. Under this treatment marked amelioration of the scorbutic symptoms will be manifest within a few days, while rheumatic or syphilitic changes will of course be unaffected.

